# Lipopolysaccharide-Induced Functional Alteration of P-glycoprotein in the Ex Vivo Rat Inner Blood–Retinal Barrier

**DOI:** 10.3390/ijms232415504

**Published:** 2022-12-07

**Authors:** Kiyotaka Daikohara, Shin-ichi Akanuma, Yoshiyuki Kubo, Ken-ichi Hosoya

**Affiliations:** 1 Department of Pharmaceutics, Graduate School of Medicine and Pharmaceutical Sciences, University of Toyama, 2630 Sugitani, Toyama 930-0194, Japan; 2 Laboratory of Drug Disposition and Pharmacokinetics, Faculty of Pharma-Sciences, Teikyo University, Kaga 2-11-1, Tokyo 173-8605, Japan

**Keywords:** inner blood–retinal barrier, P-glycoprotein, lipopolysaccharide, toll-like receptor 4, retinal capillaries

## Abstract

At the inner blood–retinal barrier (BRB), P-glycoprotein (P-gp) contributes to maintaining the homeostasis of substance concentration in the retina by transporting drugs and exogenous toxins from the retina to the circulating blood. Under inflammatory conditions, P-gp activities have been reported to be altered in various tissues. The purpose of this study was to clarify the alterations in P-gp activity at the inner BRB due to lipopolysaccharide (LPS), an inflammatory agent, and the molecular mechanisms of the alterations induced by LPS. Ex vivo P-gp activity was evaluated as luminal accumulation of 7-nitro-2,1,3-benzoxadiazole-cyclosporin A (NBD-CSA), a fluorescent P-gp substrate, in freshly prepared rat retinal capillaries. The luminal NBD-CSA accumulation was significantly decreased in the presence of LPS, indicating that P-gp activity at the inner BRB is reduced by LPS. This LPS-induced attenuation of the luminal NBD-CSA accumulation was abolished by inhibiting toll-like receptor 4 (TLR4), a receptor for LPS. Furthermore, an inhibitor/antagonist of tumor necrosis factor receptor 1, endothelin B receptor, nitric oxide synthase, or protein kinase C (PKC) significantly restored the LPS-induced decrease in the luminal NBD-CSA accumulation. Consequently, it is suggested that the TLR4/PKC pathway is involved in the reduction in P-gp function in the inner BRB by LPS.

## 1. Introduction

P-glycoprotein (P-gp, *mdr1a/1b*) is known as a drug efflux transporter which is driven by hydrolysis of intracellular adenosine triphosphate [1]. It has been reported that P-gp accepts various organic drugs, such as anticancer agents, cardiac drugs, Ca^2+^ channel blockers, and immunosuppressants [2]. In the retina, P-gp is localized on the luminal membrane of the inner blood–retinal barrier (BRB), which is formed by retinal capillary endothelial cells [3]. Several studies have indicated functional roles of P-gp at the inner BRB in restriction of retinal drug distribution. We have reported that the in vivo distribution of [^3^H]digoxin, a substrate of P-gp, to the rat retina is significantly increased by the co-administration of the other unlabeled P-gp substrate, verapamil [4]. In addition, the in vivo retinal distribution of [^3^H]digoxin in *mdr1a* knockout rats has been reported to be significantly greater than that in wild-type rats [5]. In bovine retinal endothelial cells, it is suggested that intracellular accumulation of the other substrate of P-gp, taxol, is markedly increased by P-gp-4008, a P-gp-selective antagonist [6]. Furthermore, Tajima et al. have demonstrated the ex vivo retina-to-blood transport mediated by P-gp using freshly prepared rat retinal capillaries with a fluorescent P-gp substrate, 7-nitro-2,1,3-benzoxadiazole-cyclosporin A (NBD-CSA) [7]. These cumulative lines of evidence show the importance of P-gp at the inner BRB to understand the retinal substance distribution.

It has been reported that expression and function of P-gp are changed under several pathological conditions, such as inflammation [8,9]. As an inflammatory endotoxin, lipopolysaccharide (LPS) is well-known. It has been reported that LPS is an essential ingredient of the outer membrane of Gram-negative bacteria and regulates P-gp activity in several tissues. Moriguchi et al. have reported that the intestinal activity of P-gp is decreased at 24 h after LPS administration in rats [10]. Hepatic P-gp is also reported to be downregulated by LPS administration [11]. Hoshi et al. have reported that P-gp activity at the blood–brain barrier (BBB) is reduced in mice by intraperitoneal administration of LPS [12]. Taking the importance of P-gp at the inner BRB for the retinal distribution of its substrates into consideration, it is conceivable that P-gp activity at the inner BRB could be altered under inflammatory conditions induced by the elevated levels of LPS, resulting in changes in the retinal concentrations of substances.

During diabetes mellitus (DM), the blood concentration of LPS, which is derived from the gut, has been reported to be increased [13]. Since the inflammation which is induced by the elevated LPS levels is considered to be involved in the progression of diabetic retinopathy (DR), a serious retinal disease [14], mechanisms of LPS-related DR pathogenesis should be understood. It has been reported that P-gp accepts endogenous compounds which are involved in the pathogenesis of DR [15,16]. Hence, it is hypothesized that P-gp at the inner BRB is affected by increased LPS concentration in the circulating blood of diabetic patients and thus takes part in the onset and/or development of DR. However, it is unclear whether LPS-related regulation of P-gp activity exists in the inner BRB.

The purpose of the present study was to clarify the mechanisms of altering P-gp activity at the inner BRB by LPS. We previously established a confocal imaging-based ex vivo assay to evaluate P-gp activity at the inner BRB using freshly isolated retinal capillaries from rats [7]. By using this method, we examined the P-gp activity at the inner BRB under LPS-treated conditions. Furthermore, we investigated the molecular mechanisms which are responsible for the regulation of P-gp-mediated transport at the inner BRB by LPS.

## 2. Results

### 2.1. Alterations in P-gp Activity in the Retinal Capillaries after LPS Treatment

**Figure 1:**
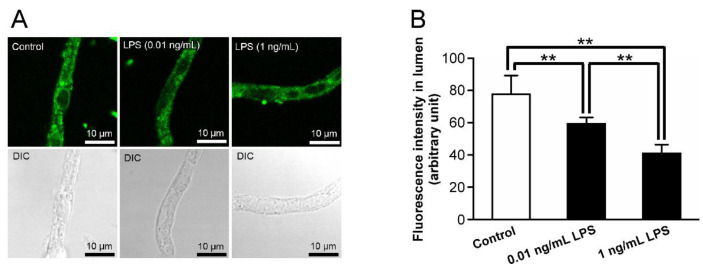
Alterations in P-gp activity in the retinal capillaries after LPS treatment. (**A**) Representative confocal images of isolated rat retinal capillaries after incubation with 4 μM NBD-CSA for 60 min. The capillaries were preincubated with ECF buffer in the absence (control) or presence of LPS at a concentration 0.01 or 1 ng/mL for 30 min. (**B**) Quantification of NBD-CSA fluorescence in the lumens of retinal capillaries. Each column represents the mean value for 10 capillaries from a single preparation; variability is given by S.D. bars. Units are expressed as arbitrary fluorescence (scale 0–255). ** *p* < 0.01, significantly different between the groups. Capillary images were captured using a TCS-SP5.

To evaluate the alterations in P-gp activity at the inner BRB by LPS treatment, NBD-CSA transport in retinal capillaries was measured after treatment with LPS. Figure 1A shows a representative confocal image of isolated rat retinal capillaries treated with or without LPS. Luminal NBD-CSA fluorescence was significantly decreased by 26% and 47% in the presence of 0.01 and 1 ng/mL LPS, respectively (Figure 1B), suggesting that the P-gp activity at the inner BRB is reduced by LPS.

### 2.2. Relationship of Toll-like Receptor 4 (TLR4) with LPS-Mediated P-gp Attenuation in the Retinal Capillaries

**Figure 2:**
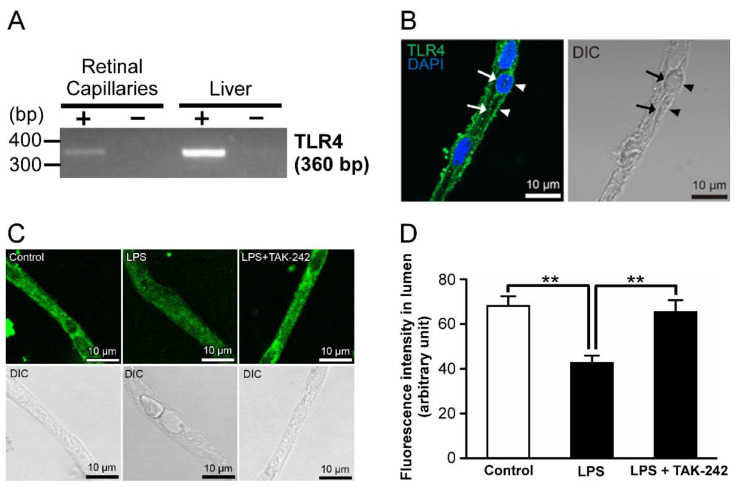
Relationship of toll-like receptor 4 (TLR4) with LPS-mediated P-gp attenuation in the retinal capillaries. (**A**) mRNA expression of TLR4 in rat retinal capillaries. RT-PCR analysis was performed in the presence (+) or absence (−) of reverse transcriptase using specific primers for TLR4. The rat liver was used as the positive control. (**B**) Localization of TLR4 proteins in rat retinal capillaries. Rat retinal capillaries were immunostained with anti-TLR4 antibodies (green). TLR4-derived immunoreactivities were observed on both the luminal (arrows) and abluminal (arrowheads) membranes of the rat retinal capillary. The nuclei were stained with DAPI (blue). Capillary images were captured using a LSM900. (**C**,**D**) Effect of TAK-242 (TLR4 inhibitor) on luminal NBD-CSA transport in rat retinal capillaries. (**C**) Representative confocal images of isolated rat retinal capillaries after incubation with 4 µM NBD-CSA for 60 min. The capillaries were preincubated with 1 ng/mL LPS in the absence or presence of TAK-242 at 1 µg/mL for 30 min. (**D**) Quantification of NBD-CSA fluorescence in the lumens of the retinal capillaries. Each column represents the mean value for 10 capillaries from a single preparation; variability is given by S.D. bars. Units are expressed as arbitrary fluorescence (scale 0–255). ** *p* < 0.01, significantly different between the groups. Capillary images were captured using a TCS-SP5.

LPS is known as a ligand for TLR4 [17]. In the RT-PCR analysis, amplified products of TLR4 mRNAs were detected in the retinal capillaries (Figure 2A). In addition, immunoreactivities of TLR4 proteins were detected on both the luminal (arrows) and abluminal (arrowheads) membranes of the retinal capillary endothelial cells (Figure 2B). With the addition of TAK-242, a TLR4 inhibitor [18], at a concentration of 1 µg/mL and LPS, luminal NBD-CSA fluorescence was increased to that in control (Figure 2C,D).

### 2.3. P-gp Attenuation by Tumor Necrosis Factor-α (TNF-α) in the Retinal Capillaries under LPS-Treated Conditions

**Figure 3:**
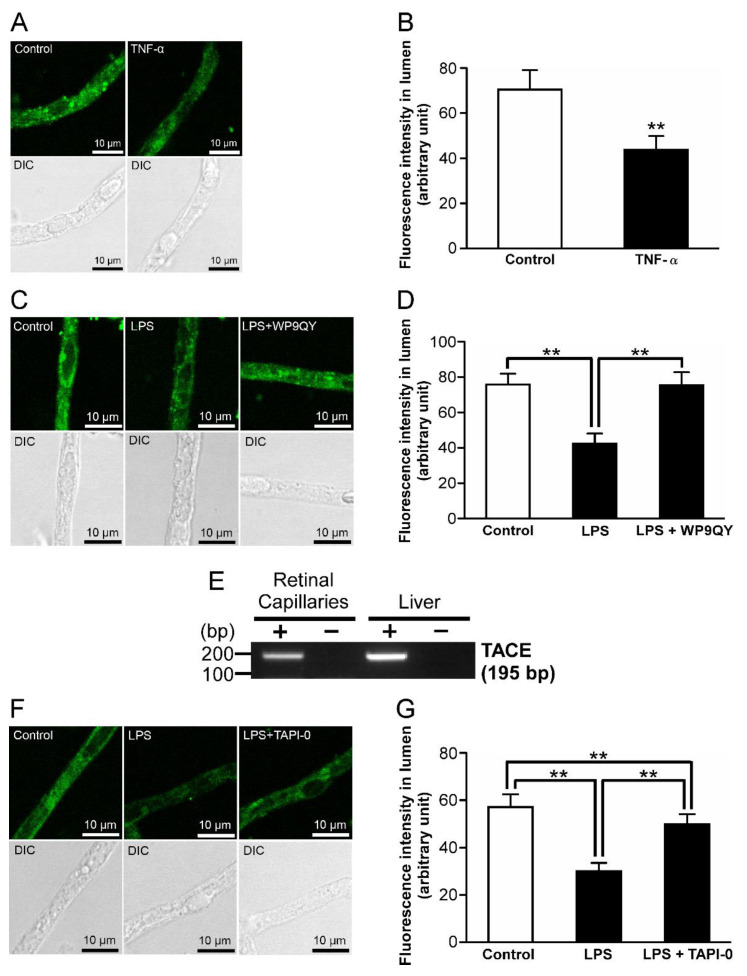
P-gp attenuation by tumor necrosis factor-α (TNF-α) in the retinal capillaries under LPS-treated conditions. (**A**,**C**,**F**) Representative confocal images of isolated rat retinal capillaries after incubation with 4 μM NBD-CSA for 60 min. (**A**) The capillaries were preincubated with ECF buffer in the absence (control) or presence of 1 ng/mL TNF-α for 30 min. (**C**,**F**) Capillaries were preincubated with 1 ng/mL LPS in the absence or presence of WP9QY (TNF-α antagonist) at 20 μM or TAPI-0 (TACE inhibitor) at 400 nM for 30 min. (**B**,**D**,**G**) Quantification of NBD-CSA fluorescence in the lumens of retinal capillaries. (**E**) mRNA expression of TACE in rat retinal capillaries. RT-PCR analysis was performed in the presence (+) or absence (−) of reverse transcriptase using specific primers for TACE. The rat liver was used as the positive control. Each column represents the mean value for 10 capillaries from a single preparation; variability is given by S.D. bars. Units are expressed as arbitrary fluorescence (scale 0–255). ** *p* < 0.01, significantly different between the groups. Capillary images were captured using a TCS-SP5.

Since TLR4 activation has been reported to induce TNF-α secretion from microvascular endothelial cells [19], it is hypothesized that TNF-α is involved in the reduction in P-gp activity as the downstream effector of TLR4 signaling. By 1 ng/mL TNF-α treatment, NBD-CSA fluorescence in the lumens of rat retinal capillaries was significantly decreased by 38% (Figure 3A,B). In addition, the reduction in luminal NBD-CSA fluorescence by LPS treatment was restored to the control level in the presence of 20 µM WP9QY, which is a TNF-α antagonist [20] (Figure 3C,D). TNF-α has been reported to be converted from a membrane-bound form to soluble form by TNF-α converting enzyme (TACE) [21]. In the RT-PCR analysis, amplified products derived from TACE mRNAs were observed in the retinal capillaries (Figure 3E). Moreover, cotreatment with 400 nM TAPI-0, a TACE inhibitor [22], and LPS significantly restored the luminal accumulation of NBD-CSA under LPS-treated conditions (Figure 3F,G).

### 2.4. Effect of an Inhibitor of TNF-Receptor 1 (TNF-R1) on LPS-Induced Attenuation of P-gp-Mediated Transport Activities in the Retinal Capillaries

**Figure 4:**
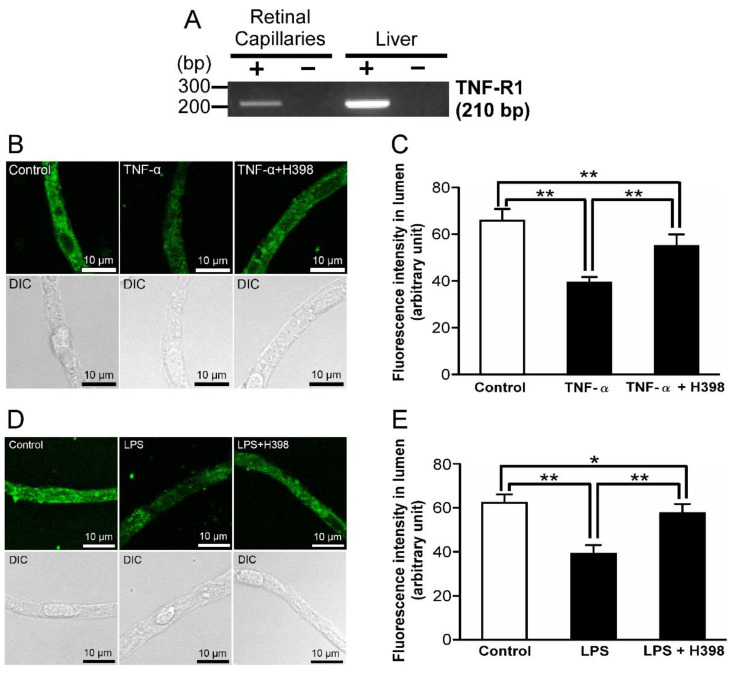
Effect of an inhibitor of TNF-receptor 1 (TNF-R1) on LPS-induced attenuation of P-gp-mediated transport activities in the retinal capillaries. (**A**) mRNA expression of TNF-R1 in rat retinal capillaries. RT-PCR analysis was performed in the presence (+) or absence (−) of reverse transcriptase using specific primers for TNF-R1. The rat liver was used as the positive control. (**B**,**D**) Representative confocal images of isolated rat retinal capillaries after incubation with 4 μM NBD-CSA for 60 min. Capillaries were preincubated with 1 ng/mL TNF-α or 1 ng/mL LPS in the absence or presence of H398 (TNF-R1 antagonist) at 10 µg/mL for 30 min. (**C**,**E**) Quantification of NBD-CSA fluorescence in the lumens of retinal capillaries. Each column represents the mean value for 10 capillaries from a single preparation; variability is given by S.D. bars. Units are expressed as arbitrary fluorescence (scale 0–255). * *p* < 0.05, ** *p* < 0.01, significantly different between the groups. Capillary images were captured using a TCS-SP5.

TNF-α has been known as a ligand for TNF-R1 [23]. In the RT-PCR analysis, TNF-R1 mRNAs were amplified in isolated rat retinal capillaries (Figure 4A). In the presence of 10 µg/mL H398, a specific TNF-R1 antagonist [24], with TNF-α or LPS, NBD-CSA accumulation in the lumens of retinal capillaries was significantly restored compared with the luminal NBD-CSA accumulation under TNF-α or LPS-treated conditions (Figure 4B–E).

### 2.5. P-gp Attenuation by Endothelin-1 (ET-1) in the Retinal Capillaries under LPS-Treated Conditions

**Figure 5:**
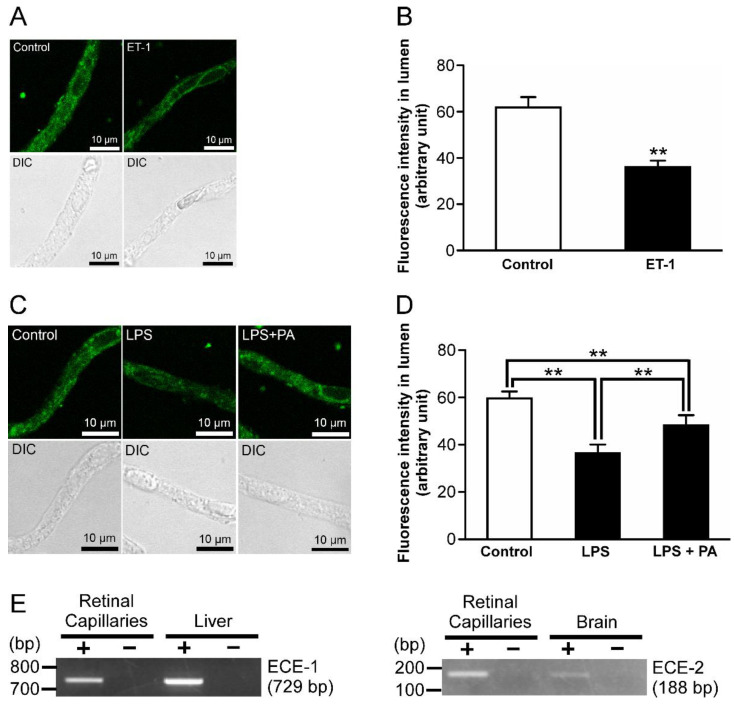
P-gp attenuation by endothelin-1 (ET-1) in the retinal capillaries under LPS-treated conditions. (**A**,**C**) Representative confocal images of isolated rat retinal capillaries after incubation with NBD-CSA at 4 μM for 60 min. (**A**) The capillaries were preincubated with ECF buffer in the absence (control) or presence of 1 nM ET-1 for 30 min. (**C**) The capillaries were preincubated with 1 ng/mL LPS in the absence or presence of PA (ECE inhibitor) at 3 μM. (**B**,**D**) Quantification of NBD-CSA fluorescence in the lumens of retinal capillaries. (**E**) mRNA expression of ECE-1 or ECE-2 in rat retinal capillaries. RT-PCR analysis was performed in the presence (+) or absence (−) of reverse transcriptase using specific primers for ECE-1 or ECE-2. The rat liver or brain was used as the positive control. Each column represents the mean value for 10 capillaries from a single preparation; variability is given by S.D. bars. Units are expressed as arbitrary fluorescence (scale 0–255). ** *p* < 0.01, significantly different between the groups. Capillary images were captured using a TCS-SP5.

ET-1 secretion has been reported as one of the downstream effectors of LPS signaling [25]. After treatment of 1 nM ET-1, luminal NBD-CSA fluorescence was significantly decreased by 42% in ET-1-treated retinal capillaries compared with the control (Figure 5A,B). In addition, the effects of LPS on luminal NBD-CSA fluorescence were significantly attenuated by the pretreatment with 3 µM phosphoramidon (PA), which suppresses ET-1 secretion via inhibiting endothelin converting enzyme (ECE) [26] (Figure 5C,D). Moreover, amplified products for mRNAs of ECE-1 and ECE-2, which are ECE isoforms, were detected in isolated retinal capillaries (Figure 5E).

### 2.6. ET_B_ Receptor-Mediated P-gp Attenuation in the Retinal Capillaries under LPS-Treated Conditions

**Figure 6:**
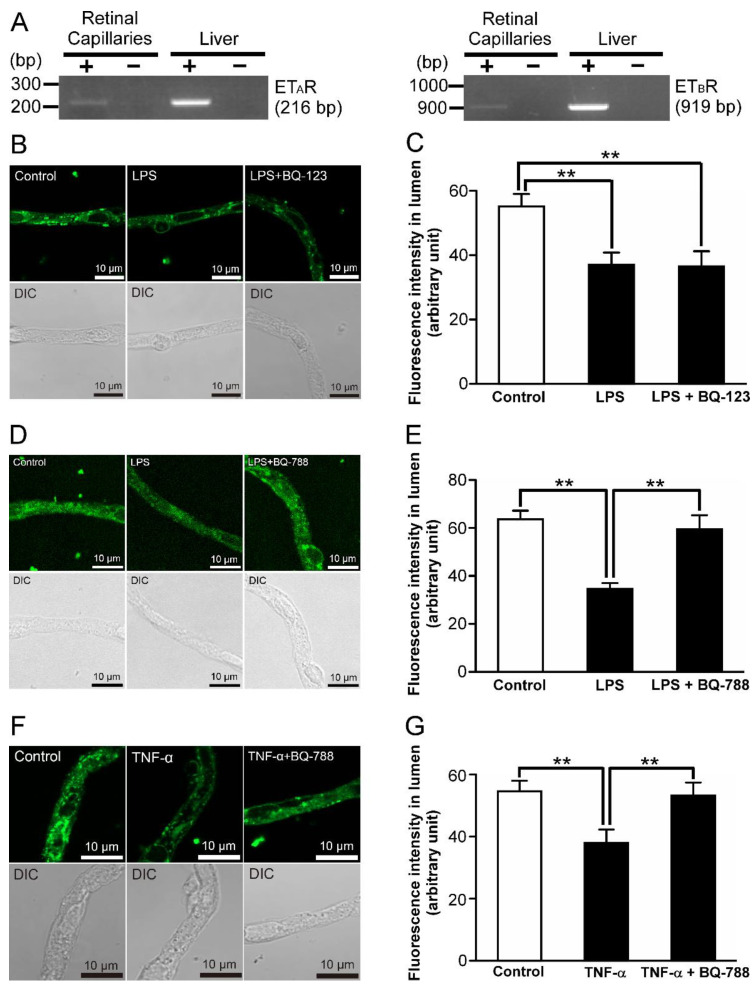
ET_B_ receptor-mediated P-gp attenuation in the retinal capillaries under LPS-treated conditions. (**A**) mRNA expression of ET_A_ receptor or ET_B_ receptor in rat retinal capillaries. RT-PCR analysis was performed in the presence (+) or absence (−) of reverse transcriptase using specific primers for ET_A_ receptor or ET_B_ receptor. The rat liver was used as the positive control. (**B**,**D**,**F**) Representative confocal images of isolated rat retinal capillaries after incubation with NBD-CSA at 4 μM for 60 min. The capillaries were preincubated with 1 ng/mL LPS or 1 ng/mL TNF-α in the absence or presence of BQ-123 (ET_A_ receptor antagonist) at 30 nM, or BQ-788 (ET_B_ receptor antagonist) at 10 nM for 30 min. (**C**,**E**,**G**) Quantification of NBD-CSA fluorescence in the lumens of retinal capillaries. Each column represents the mean value for 10 capillaries from a single preparation; variability is given by S.D. bars. Units are expressed as arbitrary fluorescence (scale 0–255). ** *p* < 0.01, significantly different between the groups. Capillary images in (**D**) were captured using a TCS-SP5, and those in (**B**,**F**) were captured using a LSM 900.

Regarding receptors for ET-1, ET_A_ and ET_B_ receptors exist [27]. In the isolated retinal capillaries, amplified products of ET_A_ and ET_B_ receptor mRNAs were detected (Figure 6A). In the presence of 30 nM BQ-123, an ET_A_ receptor antagonist [28], luminal NBD-CSA accumulation was hardly changed compared with the condition of LPS treatment (Figure 6B,C). On the other hand, 10 nM BQ-788, an ET_B_ receptor antagonist [29], abolished the effects of LPS on luminal NBD-CSA accumulation (Figure 6D,E). Furthermore, 10 nM BQ-788 showed the restoration of luminal NBD-CSA accumulation attenuated by 1 ng/mL TNF-α treatment (Figure 6F,G).

### 2.7. The Effects of Inhibitors for Nitric Oxide Synthase (NOS) and Protein Kinase C (PKC) on the Decrease in P-gp-Mediated Transport Activity in the Retinal Capillaries by LPS

**Figure 7:**
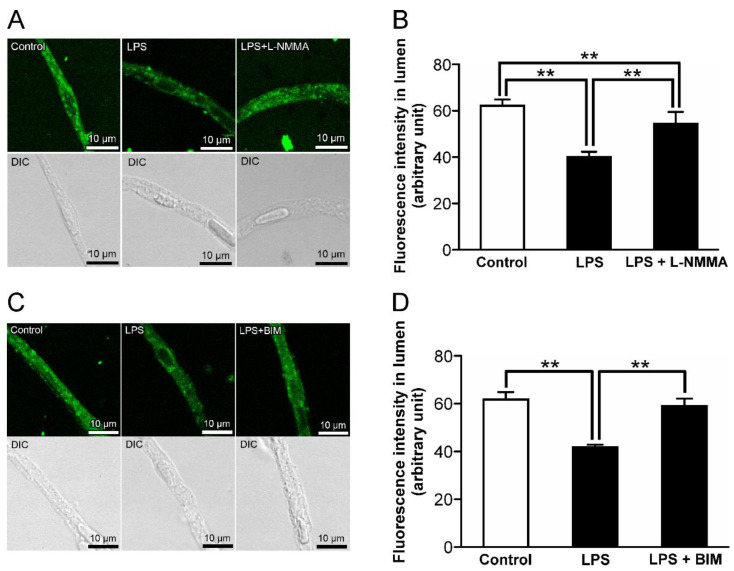
The effects of inhibitors for nitric oxide synthase (NOS) and protein kinase C (PKC) on the decrease in P-gp-mediated transport activity in the retinal capillaries by LPS. (**A**,**C**) Representative confocal images of isolated rat retinal capillaries after incubation with NBD-CSA at 4 μM for 60 min. The capillaries were preincubated with LPS at 1 ng/mL in the absence or presence of L-NMMA (NOS inhibitor) at 30 μM or BIM (PKC inhibitor) at 40 nM for 30 min. (**B**,**D**) Quantification of NBD-CSA fluorescence in the lumens of retinal capillaries. Each column represents the mean value for 10 capillaries from a single preparation; variability is given by S.D. bars. Units are expressed as arbitrary fluorescence (scale 0–255). ** *p* < 0.01, significantly different between the groups. Capillary images were captured using a TCS-SP5.

It has been reported that the stimulation of ET_B_ receptor activates NOS and PKC [30,31]. The attenuation of luminal NBD-CSA fluorescence in the rat retinal capillaries by LPS treatment was significantly restored in the presence of 30 µM *N^G^*-monomethyl-L-arginine (L-NMMA), which is a NOS inhibitor [32] (Figure 7A,B). Moreover, the effect of LPS on the luminal NBD-CSA accumulation was abolished in the presence of a PKC inhibitor, bisindolylmaleimide I (BIM; 40 nM) [33] (Figure 7C,D).

## 3. Discussion

In the present study, alteration of P-gp transport activity at the inner BRB by LPS treatment was assessed using freshly prepared rat retinal capillaries with NBD-CSA [7]. Our previous study showed that the luminal accumulation of the NBD-CSA in the retinal capillaries was decreased by 43% in the presence of 100 µM quinidine, a well-known substrate of P-gp with a Km of 5.4 µM [7,34], indicating that luminal NBD-CSA accumulation in the retinal capillaries is an index of P-gp activities at the rat inner BRB. Under LPS treatment at 1 ng/mL LPS (Figure 1), the NBD-CSA accumulation in the lumens of the retinal capillaries was significantly attenuated by 47%. Hence, it is suggested that treatment with LPS for 30 min induces functional P-gp attenuation in the inner BRB.

It has been reported that several signal cascades involve inflammatory mediator-induced regulation of P-gp. In the brain capillaries, it has been suggested that short-term exposure (30 min) to LPS induces down-regulation of P-gp activity via TLR4, TNF-α, and TNF-R1 [35]. On the other hand, Heemskerk et al. found that P-gp activities in rat renal proximal tubule epithelial cells are regulated through TLR4 activation induced by LPS and/or TNF-α, whereas it has been also indicated that the contribution of TNF-R1 is low as a downstream effector of TLR4 signaling [36]. To assess the similarity with the regulatory mechanisms of P-gp activities in other tissues by LPS, we elucidated the signal pathway for the LPS-induced P-gp attenuation at the inner BRB. The functional studies with an inhibitor of TLR4 (Figure 2C,D), which is a receptor for LPS, indicate that LPS reduces the P-gp activity at the inner BRB by acting through TLR4. Since previous in vitro and in vivo studies indicated up-regulation of TNF-α release from retinal endothelial cells by LPS [37,38], it is conceivable that secreted TNF-α takes part in TLR4-induced P-gp attenuation in the inner BRB. Indeed, LPS signaling to P-gp was inhibited by the coexistence of a TNF-α antagonist (Figure 3C,D). Furthermore, an inhibitor/antagonist for TACE (Figure 3F,G), which induces extracellular TNF-α release, or TNF-R1 (Figure 4D,E), a receptor for TNF-α, significantly restored the decrease in luminal NBD-CSA accumulation by LPS. Thus, it is suggested that LPS stimulated TNF-α release from the capillaries through TACE activation, and then TNF-α reduced P-gp-mediated transport in the inner BRB by signaling through TNF-R1.

**Figure 8:**
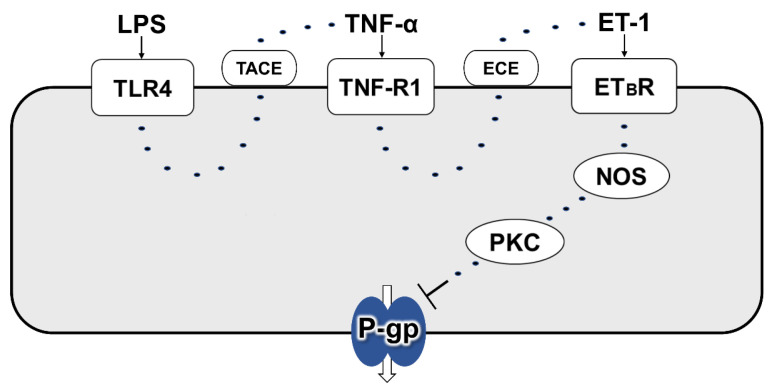
Schematic diagram of LPS-induced attenuation of P-gp activity at the inner BRB via a TLR4/PKC signaling pathway. LPS induces the release of TNF-α by acting through TLR4. The released TNF-α acts through TNF-R1, thereby stimulating the release of ET-1. The ET-1 activates an ET_B_ receptor, NOS, and PKC. Through the signaling pathway, P-gp function in the inner BRB is reduced.

In rat brain capillaries, TNF-R1 activation induced by LPS treatment for 30 min has been reported to reduce P-gp activity through ET_B_ receptor, but not ET_A_ receptor, following ET-1 release [35]. In contrast to this short-term exposure condition, long-term exposure as 6 h incubation of the capillaries to TNF-α has been reported to increase P-gp activity at the BBB by signaling through ET_A_ and ET_B_ receptors after TNF-R1-mediated ET-1 release [39]. In this study, ET-1 was suggested to reduce P-gp activity at the inner BRB (Figure 5A,B). With the cotreatment of an ECE inhibitor and LPS, the decrease in luminal NBD-CSA accumulation caused by LPS was significantly restored (Figure 5C,D). Since ECE has been reported to convert inactive big-ET-1 into biologically active ET-1 [26], this result indicates that ET-1 is involved in LPS signaling. Moreover, LPS effect was blocked by an ET_B_ receptor antagonist (Figure 6D,E), whereas an ET_A_ receptor antagonist had little effect (Figure 6B,C). This ET_B_ receptor antagonist abolished the effect of TNF-α on luminal NBD-CSA accumulation (Figure 6F,G), indicating that ET_B_ receptor is located downstream of TNF-R1 activation after LPS treatment. Taken together, it is suggested that an LPS-induced decrease in the P-gp activity at the inner BRB involves ET_B_ receptor, but not ET_A_ receptor. After the 30 min of exposure to LPS, P-gp activity in the brain capillaries is also known to be decreased via NOS and PKC [35]. On the other hand, in rat renal proximal tubule epithelial cells, it has been indicated that PKC may not be involved in P-gp regulation [36]. In rat retinal capillaries, a NOS inhibitor and a PKC inhibitor each significantly suppressed the effects of LPS on luminal NBD-CSA accumulation (Figure 7), suggesting the involvement of NOS and PKC in LPS signaling. In summary, it is suggested that LPS attenuates the P-gp activity at the inner BRB by signaling via TLR4, TNF-α, TNF-R1, ET-1, ET_B_ receptor, NOS, and PKC (Figure 8), which is consistent with what was identified in brain capillaries treated with LPS for a short time.

With long-term TNF-α treatment to the rat brain capillaries, PKC has been reported to regulate P-gp transport activity with altering P-gp expression level via NF-kB [39], which is an up-regulator of P-gp transcription in several tissues [40,41]. On the other hand, it has been indicated that several conditions of LPS treatment for less than 60 min do not change the level of NF-kB. Yu et al. have reported that NF-kB activity in vascular endothelial cells treated with 1 µg/mL LPS for 60 min was not significantly altered compared to that in the untreated cells [42]. Moreover, Hartz et al. have shown that 1 ng/mL LPS treatment for 30 min to the rat brain capillaries reduces P-gp transport activity without altering its expression level [35]. This result implies that short-term LPS treatment attenuates P-gp-mediated substrate transport without altering the level of transcriptional P-gp regulators, including NF-kB. Taking these points into consideration, it is possible that functional down-regulation of P-gp in the retinal capillaries by 1 ng/mL LPS treatment for 30 min is independent on the intracellular level of transcriptional P-gp regulators, such as NF-kB, and expression level of P-gp in the inner BRB.

In the functional studies, the LPS was treated from the retinal side in this study. Our immunostaining showed the localization of TLR4 proteins on the luminal membrane, in addition to the abluminal membrane, of the retinal capillary endothelial cells (Figure 2B). Since it is suggested that TLR4 is an initial contributor to LPS-induced P-gp attenuation at the inner BRB (Figure 2C,D), LPS in the circulating blood is considered to induce this P-gp attenuation by binding to TLR4 on the luminal membrane of the retinal capillaries. In healthy volunteers with no clinical evidence of infection, it has been reported that blood LPS levels range from 0.006 to 0.2 ng/mL; the median was reported to be 0.0143 ng/mL [43]. Since our result indicates that 0.01 ng/mL LPS attenuates P-gp activity at the inner BRB relative to control conditions (Figure 1), it is considered that P-gp activity at the inner BRB is reduced under normal conditions. In type-1 and type-2 diabetic patients, blood LPS levels have been reported to be increased by 3.36- and 1.66-fold, respectively, compared with those in non-diabetic patients [13]. In this study, luminal NBD-CSA accumulation in rat retinal capillaries treated with 1 ng/mL LPS was significantly decreased compared to that treated with 0.01 ng/mL LPS (Figure 1). Therefore, it is implied that the P-gp activity at the inner BRB is reduced in association with the concentration of LPS by 1 ng/mL. Further in vivo studies are needed to elucidate a correlation of P-gp function in the inner BRB with LPS concentration in the circulating blood. Nevertheless, it is possible that the distribution of P-gp substrates to the retina is increased during DM with elevated blood levels of LPS, thereby disrupting the homeostasis of substance concentration in the retina.

## 4. Materials and Methods

### 4.1. Animal Studies

Male Wistar/ST rats 200 g in bodyweight were obtained from Japan SLC (Hamamatsu, Shizuoka, Japan) and maintained in a controlled environment: humidity of 40–50%, 12/12 h dark/light cycle, and ~23 °C.

### 4.2. Reagents and Supplies

BIM, bovine serum albumin (BSA), BQ-788, 4′,6-diamidino-2-phenylindole (DAPI), ET-1, Ficoll^®^ PM 400 (Ficoll), LPS from *Escherichia coli* 0111:B4, TAK-242, and WP9QY were purchased from Merck (Darmstadt, Germany). H398 and TNF-α were obtained from Enzo Life Science (Farmingdale, NY, USA) and FUJIFILM Wako Chemicals (Tokyo, Japan), respectively. TAPI-0 and anti-TLR4 antibodies were purchased from Santa Cruz Biotechnology (Santa Cruz, CA, USA). BQ-123 and PA were obtained from MedChemExpress (Monmouth Junction, NJ, USA) and AG Scientific (San Diego, CA, USA), respectively. L-NMMA was purchased from Dojindo (Kumamoto, Japan). NBD-CSA was synthesized following our previous report by Tajima et al. [7]. All other reagents were commercially obtained.

### 4.3. Isolation of Retinal Capillaries

Retinal capillaries were freshly isolated by density gradient centrifugation and serial filtration with nylon-meshes from the rat retinae as described previously [7]. The prepared retinal capillaries were suspended on ice with the isolation buffer, which consisted of 137 mM NaCl, 10 mM D-glucose, 8.1 mM Na_2_HPO_4_, 2.7 mM KCl, 2.0 mM sodium pyruvate, 1.8 mM CaCl_2_, 1.5 mM KH_2_PO_4_, 0.65 mM MgCl_2_, and 1% BSA at pH 7.4.

### 4.4. P-gp Transport Assay

Freshly isolated rat retinal capillaries were spread onto glass coverslips and incubated with the reagents such as LPS for 30 min. After the adhesion onto the coverslips, the capillaries were incubated for 60 min at 25 °C with NBD-CSA at 4 µM dissolved in extracellular fluid (ECF) buffer at pH 7.4 (122 mM NaCl, 25 mM NaHCO_3_, 10 mM HEPES-NaOH, 10 mM D-glucose, 3.0 mM KCl, 1.4 mM CaCl_2_, 1.2 mM MgSO_4_, 0.40 mM K_2_HPO_4_). After the incubation, capillary images were immediately captured using a TCS-SP5 confocal laser microscope (Leica, Heidelberg, Germany) equipped with an HCX PL APO lambda blue 63 × 1.2 water objective lens or a LSM900 confocal microscopy with Airyscan 2 (Carl Zeiss, Oberkochen, Germany) under the 63 × 1.4 oil objective lens. Fluorescence of NBD-CSA in the capillary lumens was measured following the previous report [7] using ImageJ 1.52u software (Wayne Rasband, National Institutes of Health, Bethesda, MD, USA). By subtracting background fluorescence from fluorescence measured in the capillary lumens, luminal fluorescence was quantitated, and we averaged the values per capillary. As described in the previous reports [7], the level of luminal NBD-CSA fluorescence indicates the activity of P-gp in the capillaries.

### 4.5. mRNA Expression Analysis

Using TRIzol reagent (Thermo Fisher Scientific, Waltham, MA, USA), total RNA in the rat retinal capillaries, liver, and brain was isolated. The isolated RNAs were purified using the RNeasy Micro kit (QIAGEN, Hilden, Germany) following the manufacturer’s protocol. Using the isolated total RNA and oligo dT primers, complementary DNA was synthesized by ReverTra Ace (TOYOBO, Osaka, Japan) or SuperScript™ IV Reverse Transcriptase (Thermo Fisher Scientific). For the qualitative expression of mRNA, polymerase chain reaction (PCR) using ExTaq polymerase (TaKaRa Bio, Shiga, Japan) was performed with the primers shown in Table 1 under the following conditions: 35 cycles of 94 °C for 30 s, 60 °C for 30 s (TLR4: 63 °C for 30 s), and 72 °C for 45 s. PCR products were separated by electrophoresis on an ethidium bromide-containing 2–3% agarose gel. The resolved products were visualized using ultraviolet light.

### 4.6. Immunostaining

The isolated retinal capillaries were adhered on glass coverslips and then fixed at room temperature for 20 min with 3% paraformaldehyde/0.25% glutaraldehyde in Dulbecco’s phosphate-buffered saline (DPBS) which is composed of 137 mM NaCl, 2.7 mM KCl, 8.1 mM Na_2_HPO_4_, and 1.5 mM KH_2_PO_4_ at pH 7.4. The capillaries were briefly washed and treated with 0.5% Triton X-100 dissolved in DPBS for 30 min. The permeabilized capillaries were blocked with normal goat serum at a concentration of 10% (Nichirei Biosciences, Tokyo, Japan) and subsequently incubated overnight with primary antibodies against TLR4 (4 µg/mL) at 4 °C. After the reaction with anti-mouse Alexa Fluor 488-conjugated secondary IgG (1:1000; Thermo Fisher Scientific) for 90 min at 37 °C, nuclei were stained with 1 µM DAPI for 20 min. Photographs were taken using a LSM900 confocal microscope with Airyscan 2.

### 4.7. Statistical Analyses

The data are presented as the mean ± standard deviation (S.D.). Statistical differences between the means of two or more than two groups were determined using an unpaired two-tailed Student’s *t*-test or one-way analysis of variance followed by Tukey’s test, respectively.

## 5. Conclusions

The present study indicated that LPS reduces P-gp-mediated transport at the inner BRB. Furthermore, it was suggested that this effect of LPS on the P-gp activity is mediated by signaling via TLR4, TNF-α, TNF-R1, ET-1, ET_B_ receptor, NOS, and PKC. As endogenous P-gp substrates related to the pathogenesis of DR [15], endocannabinoids such as anandamide are known through inducing expression of various inflammatory mediators [16]. It is considered that a functional decrease in P-gp at the inner BRB increases the retinal concentration of P-gp substrates including endocannabinoids. Therefore, this P-gp decrease in the inner BRB by LPS which is elevated during DM is expected to participate in exacerbation of the retinal pathogenesis. In this regard, it is anticipated that the antagonism of the signal pathway which is responsible for P-gp attenuation by LPS leads to the restoration of P-gp function in the inner BRB and thus prevention of DR pathogenesis related to LPS. The signaling pathway identified in the present study would help to understand the P-gp activity at the inner BRB under pathological conditions and consider appropriate pharmacotherapies.

## Figures and Tables

**Table 1:** Primer sequences.

Molecules	Gene Bank Accession No.	Primer Sequence (Upper, Sense Primer; Lower, Antisense Primer)	Product Size (bp)
TLR4	NM_019178	5′-CAT GTC CAT CGG TTG ATC T-3′ 5′-ACT TGG CAG AGC CAA CTG ACC AAA G-3′	360
TACE	NM_020306	5′-GAG CCA TCT GAA GAG TTT GTC CGT C-3′ 5′-CCA CGA GGT GTT CCG GTA TAT GTC A-3′	195
TNF-R1	NM_013091	5′-CTG CCA CGC AGG ATT CTT TCT AAG C-3′ 5′-GGA TAT CGG CAC AGT AGA CTG ATG C-3′	210
ECE-1	NM_053596	5′-GAG GAT CTG GTG GAC TCA CTC TCC-3′ 5′-CGT CTT CAG GTA ATA GTC TCT-3′	729
ECE-2	NM_001002815	5′-TTA ACC GTA CGG AAC CAA GC-3′ 5′-GCC AAG GGC ATC ATC TGT AT-3′	188
ET_A_R	NM_012550	5′-GAA GTC GTC CGT GGG CAT CA-3′ 5′-CTG TGC TGC TCG CCC TTG TA-3′	216
ET_B_R	NM_017333	5′-AGC TGG TGC CCT TCA TAC AGA AGG C-3′ 5′-TGC ACA CCT TTC CGC AAG CAC G-3′	919

TLR4, toll-like receptor 4; TACE, TNF-α converting enzyme; TNF-R1, TNF-receptor 1; ECE-1, endothelin converting enzyme-1; ECE-2, endothelin converting enzyme-2; ET_A_R, ET_A_ receptor; ET_B_R, ET_B_ receptor.

## Data Availability

The data of this study are available from the corresponding author upon reasonable request.
